# Can you imagine this? Imagined contact as a strategy to promote positive intergroup relations

**DOI:** 10.3389/fpsyg.2023.1226503

**Published:** 2023-06-29

**Authors:** Lipaz Shamoa-Nir, Irene Razpurker-Apfeld

**Affiliations:** Department of Behavioral Sciences, Zefat Academic College, Safed, Israel

**Keywords:** imagined contact, intergroup relations, majority/minority, prejudice, religious symbols, review

## Abstract

In comparison to the extensive body of research on intergroup contact, which encompasses predictors, outcomes, and implications, there has been relatively little attention given to the role of imagined contact with diverse ethno-religious out-groups. This gap particularly exists in understanding the factors that contribute to the effectiveness of imagined contact interventions. This article aims to address this gap by presenting current research on the predictors and consequences of imagined contact. We offer an overview of the circumstances in which imagined contact proves beneficial; while focusing on research that holds relevance for designing interventions and policies promoting contact between culturally and religiously diverse groups and individuals. We also acknowledge the existing limitations within this field of study and propose potential direction for future research.

## Introduction

Interpersonal contact has been hypothesized to be a valuable means of enhancing relations between groups (Allport, 1954). Indeed, the effectiveness of face-to-face contact in reducing negative intergroup attitudes has been demonstrated in various contexts (Pettigrew and Tropp, 2006). Originally, intergroup contact interventions were designed as in-person meetings, emphasizing shared goals (Allport, 1954). However, a recent meta-analysis suggests that, in accordance with the extended contact hypothesis, extended contact can positively influence intergroup attitudes even without direct personal contact or friendship (Zhou et al., 2019). Recognizing the challenges associated with establishing direct contact between certain groups, more theoretical research and practical solutions are needed to improve group relations through alternative means. Non-direct contact refers to situations where personal contact with out-group members is not required. It may involve being aware of in-group members interacting with out-group members, encountering out-group representation through media, or engaging in mentally simulated positive contact with out-group members. In this article, we primarily focus on the latter form of non-direct contact known as imagined contact (Crisp and Turner, 2012).

Research suggests that imagined contact has an impact on intergroup relationships and future behaviour (Turner and Crisp, 2010). Imagining pleasant intergroup contact with an out-group member reduces intergroup anxiety (Razpurker-Apfeld and Shamoa-Nir, 2020). Moreover, perceiving imagined contact as cooperative is crucial for reducing prejudice and anxiety, and increasing empathy and trust (Kuchenbrandt et al., 2013). Indirect contact, on the whole, is effective in promoting positive attitudes towards a variety of participants and out-group targets, including adults (Zhou et al., 2019) and children (Nasie et al., 2022). Even among adolescents, forms of indirect contact impact prejudice-related variables by promoting social-analytical thinking, using imagined scenarios to deal with cognitive distortions related to ethnic beliefs (D’Errico et al., 2023).

However, the attainment of expected positive outcomes through imagined contact can be hindered by various intergroup attitudes that impede the quality and quantity of interpersonal communication and relationships. Thus, clarifying the conditions under which indirect contact can be highly effective is crucial for intergroup contact literature (Stephan, 2014). This is particularly important given the diverse methods and manipulations employed in this field. For example, while some studies provided participants with information about the imagined counterpart using specific scenarios, others manipulated the biographies of out-group members without examining it in the context of imagined contact (Yogeeswaran and Dasgupta, 2014). Consequently, the objective of this review is to explore significant factors associated with imagined contact and highlight variables and conditions that should be taken into consideration in future investigations.

## How can imagined contact promote positive interactions with out-group members?

Research has consistently demonstrated the positive impact of imagined contact on intergroup attitudes. A meta-analysis has provided strong evidence for the effectiveness of positive imagined contact (Miles and Crisp, 2014). Studies have shown the effectiveness of imagined contact and related non-direct contact in improving intergroup attitudes and relations among individuals spanning from childhood through adulthood (Turner and Crisp, 2010; Zhou et al., 2019; Nasie et al., 2022; D’Errico et al., 2023). These studies, among others, have revealed improvements across a wide range of outcome variables, including out-group trust (Turner et al., 2013), contact self-efficacy (Stathi et al., 2011), humanization (Prati and Loughnan, 2018), positive behaviours toward out-group members (Turner and West, 2012), and ethnic moral disengagement (D’Errico et al., 2023).

The study of expectations and intentions regarding future interactions with out-group members has garnered considerable attention when exploring the implications of both actual and imagined contact in intergroup interactions (Husnu and Crisp, 2010). Research has found that indirect contact encourages direct contact between group members, particularly when individuals mentally simulate certain behaviours that subsequently increase their intentions to engage in those behaviours in the future (Wölfer et al., 2018). Furthermore, exposure to cooperative scenarios has been shown to generate positive expectations for social interactions with the imagined out-group (Kuchenbrandt et al., 2013). Additionally, studies have demonstrated that inventing scenarios strengthens actual intentions to act on the imagined plans, such as donating blood (Anderson, 1983) or engaging in contact with an out-group member (Husnu and Crisp, 2010).

Recent research on the extended contact hypothesis has revealed that simply knowing that in-group members have cross-group friends can significantly enhance attitudes toward the corresponding out-group (Zhou et al., 2019), underscoring the influence of perception on contact outcomes. In addition, a recent study has indicated the importance of the characteristics of out-group counterparts in reducing out-group prejudice and intergroup anxiety following imagined scenarios (Razpurker-Apfeld and Shamoa-Nir, 2020). However, this study also suggests that the impact of imagined contact on improving interpersonal interactions with out-group members is not always guaranteed and should be examined considering specific factors that can result in mixed effects and, in some cases, even negative social outcomes.

## Future research directions

The field of imagined contact research has yielded valuable insights through studies involving diverse groups in various conflict locations (Stathi et al., 2011; Husnu and Paolini, 2018). However, there is a need to further explore the determinants and nuances that contribute to successful imagined contact. In planning future research, it is important to consider the following suggestions and challenges to enhance the literature:

### History of intergroup relations (status, majority-minority relations, and group affiliation)

The social structure of groups involved in imagined contact cannot be overlooked when considering intentions and future interactions with different out-group members. Positive impact of imagined contact is generally stronger for majority groups compared to minorities (Bagci et al., 2018). Intergroup anxiety influences contact differently depending on group status (Vedder et al., 2017). Pettigrew and Tropp (2006) proposed that minority groups, due to their lower societal status, may anticipate discrimination, hindering positive outcomes. Disadvantaged group members may also experience heightened emotions and anxiety in certain contact situations, fearing legitimization of their lower status (Ron et al., 2017). Moreover, the evidence concerning settings that have been found to promote intercultural contact and reduce negative attitudes (Tawagi and Mak, 2015), being affiliated with a certain status group cannot be ignored; because in similar environments low status group members may still feel dominated by the high-status group, for example when helpful contact is considered (Halabi et al., 2016).

Group affiliation significantly impacts attitudes towards out-group members, with stronger identification correlating with more negative attitudes (Kaiser and Pratt-Hyatt, 2009). The intensity of intergroup anxiety is determined not only by group affiliation (Ron et al., 2017) or personal factors such as prior experience with out-group members and expectations of negative consequences (Plant and Devine, 2003) and in-group identification (Bizman and Yinon, 2001); but also, by situational factors, including perceived distance. For example, when threatening out-group members appeared to be physically close, individuals who were highly identified with their in-group chose to sit farther away from these members (Xiao et al., 2016). Positive outcomes have been observed when participants imagine engaging in a pleasant conversation with an out-group individual. It is important to consider planning interventions that incorporate imaginary interactions also in contexts of rivalry and intergroup conflicts.

### Physical closeness

Physical proximity to an imagined out-group person is a significant factor within imagined contact process. Positive contact conditions involve envisioning the out-group person in close physical proximity, while in control conditions the out-group person is utterly absent (Bagci et al., 2018). The threat hypothesis suggests that threatening out-group members are represented as physically close to the alert observer (Fini et al., 2018). Physical proximity between members of competing groups is associated with the risk of losing status, increased hostility, and a desire to maintain distance to restore safety boundaries (Xiao et al., 2016). Imagined contact encourages participants to imagine an out-group person in close proximity to themselves (Bagci et al., 2018), but the presence of a threatening out-group member at a close distance might amplify hostility and a desire to remove threat (Xiao et al., 2016). Future research should explore the optimal distance that balances the positive impact of imagined contact with the potential negative effects of imagined physical proximity on intergroup attitudes.

### Perceived typicality of out-group

Perceived typicality of out-group members refers to how closely a person aligns with the characteristics, appearance, language, thinking and behaviors associated with their group. Research has shown increased effects when participants imagined positive-cooperative contact as opposed to only positive contact (Kuchenbrandt et al., 2013). However, it remains unclear how perceived typicality of the out-group member interacts with the type of imagined contact and its impact on out-group attitudes. This is an important factor to consider as it may predict intergroup attitudes, but its influence in the context of imagined contact, particularly when the out-group member embodies visible stereotypes (Kende and McGarty, 2019), is not well understood.

In the context of racial groups, research has shown that White individuals tend to react more negatively to Black individuals with Afrocentric physical features compared to those who deviate from the prototypical look, suggesting the activation of group stereotypes based on physical features (Blair et al., 2002). Additionally, exposure to specific group content, such as clothing, religious symbols and concepts, has been found to serve as threat-cues and influence attitudes towards out-group members (Shamoa-Nir & Razpurker-Apfeld, 2020; Razpurker-Apfeld and Shamoa-Nir, 2021). This suggests that even within the context of imagined contact the typicality of out-group members envisioned with their group symbols may evoke threat and negative attitudes.

Taken together, imagined positive contact may lead to more positive attitudes when the out-group person looks casual than when he includes prominent visual cues categorizing him as a typical member of his group. Experimental studies have shown that priming of religious concepts affected out-group attitudes (Shamoa-Nir and Razpurker-Apfeld, 2020) and pro-social behavior among religious and non-religious people (Ahmed and Salas, 2008). Hence, further research should aim to gain a better understanding of the features of the out-group person involved in imagined contact and consider different types of out-group members to examine the influence of imagined contact on their attitudes and interactions.

### Intergroup anxiety

Intergroup anxiety, the feeling of personal threat during interactions with out-group members, plays a crucial role in the relationship between intergroup contact and reduced prejudice (Pettigrew and Tropp, 2006). By interacting with out-group members, individuals can alleviate their fear and anxiety perceived from the out-group, leading to more positive evaluations. Intergroup anxiety negatively predicts contact with out-group members and is associated with avoidance of intergroup interactions (Plant and Devine, 2003; Shook and Fazio, 2011; Stephan, 2014). Higher intergroup anxiety also predicts more negative expectations from future interactions with the out-group. Previous research has explored the interactive effects of imagined contact and the characteristics of the imagined person on intergroup anxiety and prejudice, highlighting the importance of individual’s group affiliation in imagined contact (Razpurker-Apfeld and Shamoa-Nir, 2020). The role of group anxiety in facilitating direct contact through indirect contact has also been emphasized (Wölfer et al., 2018). However, further investigation is needed to understand the extent and conditions under which imagined contact can effectively reduce intergroup anxiety.

## Conclusion

This article sheds light on several aspects involved in imagined contact which may influence intergroup attitudes. While providing theoretical contributions to social psychology and social cognition, further empirical research is needed, particularly in diverse societies. The findings presented in this article offer a foundation for understanding imagined contact expectancies and can contribute to improving intergroup relations globally. Hopefully, these insights would inspire further growth and enrichment of the scientific literature in this field.

## Author contributions

All authors listed have made a substantial, direct, and intellectual contribution to the work and approved it for publication.

## Funding

This work was supported by the Zefat Academic College under Grant 27-2021.

## Conflict of interest

The authors declare that the research was conducted in the absence of any commercial or financial relationships that could be construed as a potential conflict of interest.

## Publisher’s note

All claims expressed in this article are solely those of the authors and do not necessarily represent those of their affiliated organizations, or those of the publisher, the editors and the reviewers. Any product that may be evaluated in this article, or claim that may be made by its manufacturer, is not guaranteed or endorsed by the publisher.
